# INFLAMMATORY BIOMARKERS DIFFER AMONG HOSPITALIZED VETERANS INFECTED WITH COVID-19 VARIANTS

**DOI:** 10.1093/geroni/igac059.2978

**Published:** 2022-12-20

**Authors:** Catherine Park, Benjamin Seligman, Bret Hicken, Shahriar Tavakoli-Tabasi, Amir Sharafkhaneh, Christopher Amos, Andrew Chou, Javad Razjouyan

**Affiliations:** Veterans Affairs, Houston, Texas, United States; David Geffen School of Medicine at UCLA, Los Angeles, California, United States; Veterans Affairs, Salt Lake City, Utah, United States; Baylor College of Medicine, Houston, Texas, United States; Baylor College of Medicine, Houston, Texas, United States; Baylor College of Medicine, Houston, Texas, United States; Baylor College of Medicine, Houston, Texas, United States; Veterans Affairs, Houston, Texas, United States

## Abstract

The SARS-CoV-2 infection pathogenesis reported a complex interplay between virus and host immune response. In this study, we hypothesized that Omicron variant causes less inflammation and cytokine release than Delta and Alpha variants as measured by the first laboratory biomarkers during hospitalization. This is a retrospective cohort study of veterans who tested positive for COVID-19 and were hospitalized at the Veterans Health Administration due to COVID-19 infection. We defined three groups of patients based on highly likely period representing variants: Alpha (12/01/2020-05/31/2021), Delta (09/01/2021-11/30/2021), and Omicron (02/01/2022-07/11/2022). The risk of abnormality levels in biomarkers (C-reactive protein [CRP], ferritin, albumin, alanine aminotransferase [ALT], aspartate transaminase [AST], and lactate), as well as in-hospital mortality, were calculated using logistic regression adjusted by age, sex, body-mass-index, race, Charlson comorbidity index, VA frailty index, and vaccine record status. Of 414,213 Veterans tested for COVID-19, 74,342 Veterans (age: 67.8±14.3 years, BMI: 29.4±7.1 kg/m2) met the criteria: Alpha, 18,159 (34.4%); Delta, 23,414 (44.4%); Omicron, 11,207 (21.2%). Compared with Omicron, we observed significantly higher odds of abnormality levels in CRP (Alpha, adjusted odds ratio (aOR)=1.38; Delta, aOR=1.74), ferritin (Alpha, aOR=2.02; Delta, aOR=2.33), albumin (Alpha, aOR=1.16; Delta, aOR=1.15), ALT (Alpha, aOR=1.13; Delta, aOR=1.12), AST (Alpha, aOR=1.31; Delta, aOR=1.57), and lactate (Alpha, aOR=1.62; Delta, aOR=2.27) as well as mortality (Alpha, aOR=2.19; Delta, aOR=2.95). Veterans infected with Omicron showed less severe biomarkers’ responses compared to Alpha and Delta and lower mortality risk. Understanding the biomarkers’ responses of each patient across the different variants could be used to enhance acute patients’ management.

